# A new treatment for sarcoma extracted from combination of miRNA deregulation and gene association rules

**DOI:** 10.1038/s41392-023-01470-z

**Published:** 2023-06-05

**Authors:** José Manuel García-Heredia, Marco Pérez, Eva M. Verdugo-Sivianes, María M. Martínez-Ballesteros, Sara M. Ortega-Campos, Amancio Carnero

**Affiliations:** 1 Instituto de Biomedicina de Sevilla (IBIS)/HUVR/Universidad de Sevilla/CSIC, Sevilla, Spain; 2 Departamento de Bioquímica Vegetal y Biología Molecular, Universidad de Sevilla, Sevilla, Spain; 3 CIBERONC, IS Carlos III, Madrid, Spain; 4 Departamento de Anatomía Patológica, HUVR, Sevilla, Spain; 5 Departamento de Lenguajes y Sistemas Informáticos, Escuela Superior de Ingeniería Informática, Universidad de Sevilla, Sevilla, Spain

**Keywords:** Sarcoma, Non-coding RNAs, Cancer therapy

**Dear Editor**

Sarcomas are a group of heterogeneous mesodermic rare tumors with high incidence in children, reaching up to 20% of neoplasms. Standard treatment for sarcomas is surgical resection, and only some patients are treated with chemotherapy and/or radiation therapy. The 5-year relative survival rate for patients with metastatic sarcoma is only 15%.^1^ Therefore, new treatments are required to increase the survival of these patients. In recent years, microRNAs have shown promising results as prognostic and diagnostic biomarkers in multiple types of sarcoma,^2,3^ so unique miRNA patterns and miRNA profiles identification could improve diagnosis and new therapies development.^3–5^ Here, we analyzed changes in miRNA expression in sarcoma. With in-depth bioinformatics analysis, the use of association rules, and reported gene-drug associations, we established a new flow chart for treatment analysis, identifying several potential new treatments for sarcoma patients.

To search for differentially expressed miRNAs in sarcomas, we compared the expression of miRNAs in different sarcoma cell lines with the nontumor, IMR90 cell line, and between 17 sarcomas and 6 nontumor mesenchymal tissues (Fig. 1a). We identified 81 miRNAs (23 down, 49 up) (Supplementary Table 1) modified in sarcoma cells compared to the IMR90 cell line, and 51 (49 down, 2 up) miRNAs from patient’s sarcoma samples vs nontumor samples (Supplementary Table 1). Comparing both lists, we identified 16 downregulated miRNAs and 1 upregulated miRNA, hsa-miR-138-5p, which exhibited a dual effect (oncogene and tumor suppressor), therefore we focused only on the 16 downregulated miRNAs (Fig. 1a).

**Fig. 1:**
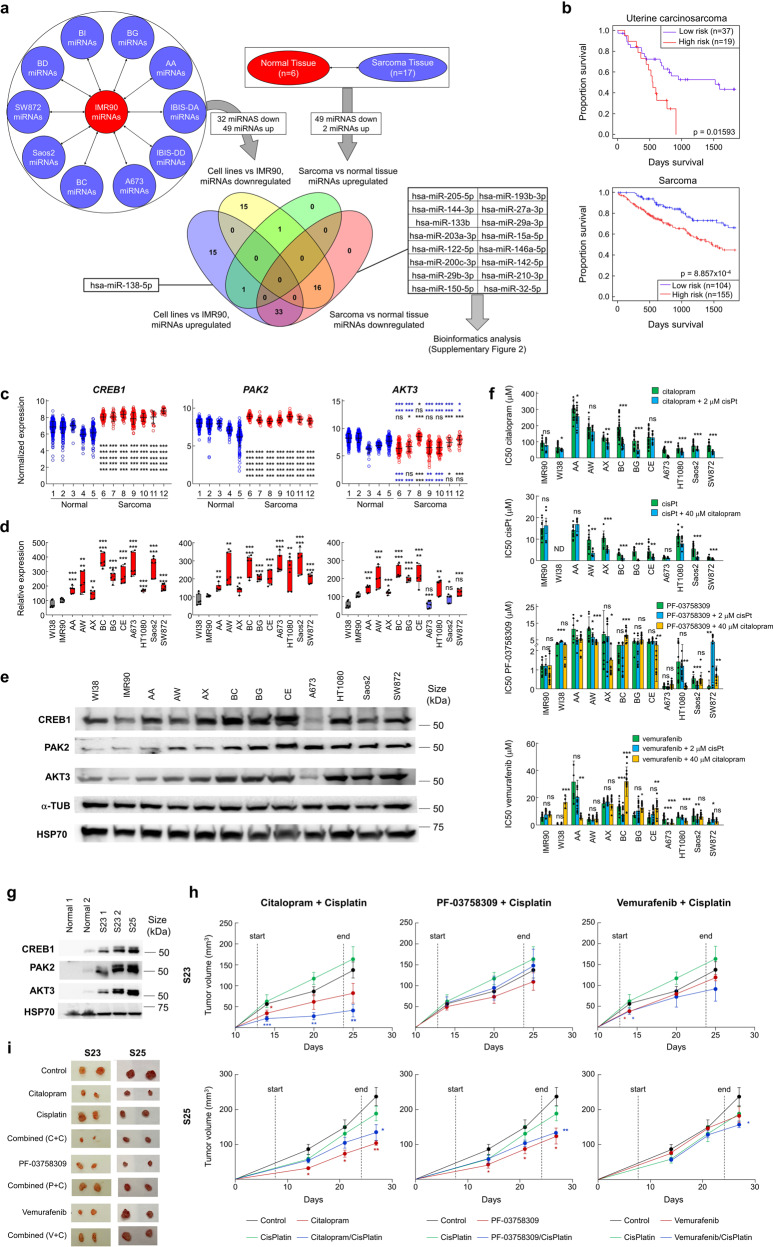
**a** Summary of the strategy to obtain a series of miRNAs of which the expression is modified in the same way in both cells in culture and tumors. Using this method, 16 downregulated miRNAs and 1 upregulated miRNA were found in both cell lines and tumor samples. **b** Patient survival based on the expression of the 16 downregulated miRNAs, observing a correlation between survival and miRNA expression. **c** Expression of *CREB1*, *PAK2* and *AKT3* in sarcoma vs. normal datasets (1: GSE7307, 2: GSE3526, 3: GSE9103, 4: GSE3307, 5: GSE1133 6: GSE34620, 7: GSE12102, 8: GSE66533, 9: GSE17679, 10: GSE142162, 11: Aqeilan dataset (R2), 12: GSE14827). *CREB1* and *PAK2* expression was significantly induced in all sarcoma datasets regarding normal tissues, with a *p*-value < 0.001 (***). Regarding *AKT3*, blue asterisks refers to downregulation and black asterisks refers to upregulation in sarcoma regarding normal datasets. **d** Expression of *CREB1*, *PAK2* and *AKT3* in sarcoma cells relative to nontumor cells, measured by qRT‒PCR. **e** Protein expression of CREB1, PAK2 and AKT3 in cells showing a general increase for all of them. HSP70 and α-TUB were used to normalize protein levels. **f** IC50 values for citalopram, cisplatin, PF-03758309, and vemurafenib, individually or combined with a second drug, showing increased sensitivity for combined treatments in sarcoma cells in relation to nontumor cells. **g** Protein expression of CREB1, PAK2 and AKT3 in PDXs, showing increased expression in nontumor samples. HSP70 was used to normalize protein levels. **h** PDXs generated in mice were treated with citalopram, PF-03758309, vemurafenib, cisplatin or a combination of cisplatin with one of the previous drugs, showing that most of the treatments produced a significant reduction in tumor volume. Start and End refer to the extension of each treatment. **i** PDX tumors were surgically extracted at the end, showing a clear reduction in volume due to treatments with citalopram, citalopram plus cisplatin, PF-03858309 and vemurafenib (only in S23). C + C: citalopram plus cisplatin; P + C: PF-03858309; V + C: vemurafenib plus cisplatin. Student’s *t* test statistical analysis of the data was performed to find significant differences (*p < 0.05; **p < 0.01; ***p < 0.001, ns non-statistical significance)

Briefly, the 16 downregulated miRNAs were correlated with target genes with increased expression in sarcoma tumors vs non-tumoral samples. GO analysis revealed association to increased metastatic capacity and low 5-year survival rate.

To choose possible treatments, we used the GSE21050 sarcoma dataset, which contains data on gene expression, metastases, and survival data. Applying the *Apriori* algorithm to the dataset, we looked for the most frequent and strongest associations between highly expressed genes and metastasis in patients with a survival time lower than 5 years. The *Apriori* algorithm identified three common genes: AKT3, CREB1, and PAK2 (Supplementary Fig. 2, Supplementary Table 5), selecting drugs correlating with these genes as biomarkers. A detailed description of the full process can be found in Supplementary materials.

*AKT3* has previously been linked to vemurafenib in melanoma cells.^6^ *CREB1* appeared connected to citalopram, an antidepressant that exerts some antimetastatic effect in cancer cells.^7,8^ Finally, *PAK2*, commonly upregulated in different tumors,^9^ was connected to its inhibitor PF-03758309.^10^ To establish a second drug for possible combined treatments, we looked for association rules between: (1) each of the three genes; (2) metastasis; and (3) other genes with common drugs (Supplementary Fig. 2). We found that each of the genes correlated with the others, allowing us to implement double treatments with the primary drugs selected (vemurafenib plus citalopram, vemurafenib plus PF-03758309, or citalopram plus PF-03758309). We also found, a high number of genes related to drugs causing DNA damage, such as cisplatin, oxaliplatin, fluorouracil, or paclitaxel (Supplementary Fig. 2). To reduce the number of combinations to test as PoC, we decided to use cisplatin as a secondary drug, as numerous genes related to *AKT3*, *CREB1* and *PAK2* appeared connected to this drug.

Next, we experimentally tested the results of our flow chart on new combinations of drugs for sarcomas.

To validate the selected genes, we analyzed their expression in different sarcoma datasets using R2. We found that the three genes were consistently deregulated in most sarcomas and could be considered general biomarkers (Fig. 1c). However, *AKT3* appeared to be downregulated specifically in Ewing sarcoma samples (datasets 6, 7, 9, 10), indicating a potential tumor-specific effect. Furthermore, we analyzed the expression of the three genes in sarcoma cells using nontumor IMR90 and WI38 cells as controls. We found a clear overexpression of CREB1, PAK2, and AKT3 (Fig. 1d, e, Supplementary Fig. 3), with the exception of AKT3 in A673, a cell line derived from Ewing sarcoma. Therefore, we analyzed cell survival after treatment with each of the considered drugs, individually or in combination. We found that, both individually and in combination, sarcoma cells showed increased sensitivity respect nontumor cells (Fig. 1f), supporting the relationship between gene expression and drug resistance. The best combinations were citalopram plus a suboptimal dose of 2 µM cisplatin, or cisplatin with a suboptimal dose of 40 µM citalopram, both of which induced a significant decrease in IC50 values for most of the sarcoma cells tested. However, combined treatment with suboptimal doses of citalopram or cisplatin with vemurafenib or PF-03758309 did not significantly decrease IC50 values for most of the sarcoma cells tested.

To test the effect of the selected drugs in vivo, alone or combined with a second drug, we used two previously described PDX sarcoma models (see M&M). We found increased expression of the three biomarkers, CREB1, AKT3, and PAK2, in PDXs compared to nontumor tissue (Fig. 1g). PDXs generated—were randomized (*n* = 6 in each group) for treatment with citalopram, PF-03758309, or vemurafenib, alone or in combinations of these drugs (citalopram + PF-03758309, citalopram + vemurafenib, or PF-03758309 + vemurafenib). Furthermore, based on connections between the first gene and a second group of genes, we used additional combinations of drugs: citalopram + cisplatin, PF-03758309 + cisplatin, vemurafenib + cisplatin, and cisplatin alone as a control. Genes related to cisplatin had more association rules than others, appearing repeatedly in our analysis, so we included cisplatin as secondary/combinatory treatment. Interestingly, in our PDX models, cisplatin alone barely had any efficacy (Fig. 1h, Supplementary Fig. 4). However, we found a significant reduction in tumor volume when citalopram was used alone or, especially, combined with cisplatin, resulting in a significant increased survival (Fig. 1I). Citalopram combined with vemurafenib or PF also showed some efficacy in our PDXs, higher in the case of S25 (Fig. 1h, i, Supplementary Fig. 4). Furthermore, PDX treatment with vemurafenib or PF-03758309 alone also caused a significant reduction in tumor volume, higher for S23 (Fig. 1h, i, Supplementary Fig. 4). The combination of these two drugs did not cause a significant reduction in tumor volume (Supplementary Fig. 4), while its combination with cisplatin also showed increased efficacy.

Here, we present a new approach for identifying treatment combinations for sarcoma. We demonstrate that identifying a list of common downregulated miRNAs in sarcoma can be used to design potential treatments. These miRNAs regulate, in a complex network of multiple nodes, a wide set of genes, many of which are commonly dysregulated in sarcoma. Gene Ontology analysis suggests an increase in metastasis. Therefore, the relationship of the miRNAs with their targets allowed us to identify biomarkers of metastases. We identified *AKT3*, *CREB1*, and *PAK2* as potential biomarkers for sarcoma metastasis, designing new individual or combined treatments, with drugs related to each gene. We found that the combination of citalopram (related to *CREB1* expression) and cisplatin, two drugs that are not currently used to treat sarcomas, showed some efficacy in vitro and in vivo. The same happened when we combined vemurafenib with citalopram. We also observed a reduced tumor size in mice, suggesting that citalopram could be used as an adjuvant drug to enhance the effects of other drugs.

We also identified high number of association rules between citalopram-related genes and 5-FU and paclitaxel-related genes, suggesting that, although not common in sarcoma, these combinations should be tested. Once the mechanics of the algorithm are established, it may be possible to identify and experimentally test many other combination therapies based on biomarker association rules for other tumor types, enabling easy drug repositioning.

## Supplementary information

Supplementary Materials

Supplementary tables

## Data Availability

The datasets used and/or analyzed during the current study are free and available from the scientific literature.
