# Supplementary material for: A new treatment for sarcoma extracted from combination of miRNA deregulation and gene association rules
Source: Signal Transduct Target Ther. 2023 Jun 5;8:231. doi: 10.1038/s41392-023-01470-z (PMC10239747; doi:10.1038/s41392-023-01470-z)

Supplementary Materials for

**A new treatment for sarcoma extracted from combination of miRNA deregulation and gene association rules**

José Manuel García-Heredia^1,2,3,*^, Marco Pérez^1,3,4^, Eva M. Verdugo-Sivianes^1,3^, María del Mar Martínez-Ballesteros^4^, Sara M. Ortega-Campos^1,3^, Amancio Carnero^1,3,*^

^1^Instituto de Biomedicina de Sevilla (IBIS), Hospital Universitario Virgen del Rocío, Universidad de Sevilla, Consejo Superior de Investigaciones Científicas, Spain

^2^Departamento de Bioquímica Vegetal y Biología Molecular, Universidad de Sevilla, Spain

^3^CIBER de Cáncer, IS Carlos III, Madrid, Spain

^4^Departamento de Anatomía Patológica, Hospital Universitario Virgen del Rocío, Spain

^5^Departamento de Lenguajes y Sistemas Informáticos, Escuela Superior de Ingeniería Informática, Universidad de Sevilla, Spain

(*) Corresponding authors: Amancio Carnero; acarnero-ibis@us.es, Telephone: (+34) 955923111. José M. García-Heredia; jmgheredia@us.es, Telephone: (+34) 955923111

Instituto de Biomedicina de Sevilla/HUVR/CSIC

Hospital Universitario Virgen del Rocío

Avda. Manuel Siurot s/n

41013, Sevilla, Spain

[acarnero-ibis@us.es](mailto:acarnero-ibis@us.es), [jmgheredia@us.es](mailto:jmgheredia@us.es)

Telephone: (+34) 955923111

**This PDF file includes:**

Detailed information of the algorithm process

Supplementary Materials and Methods

Supplementary Text

Supplementary Figures 1 to 4

Supplementary Tables 1, 6 and 7

References (supplementary references)

Uncropped WB figures

**Detailed information of the algorithm process**

To test the effect on survival of the expression changes of the 16 downregulated miRNAs, we used the available sarcoma databases on Oncomir resource, finding an association between these miRNA signature and lower survival (**Fig. 1b**). To identify the genes associated with the altered miRNAs, we used the MIENTURNET resource, using miRTarBase filtering, obtaining a multinode network in which miRNAs miR-29a-3p and miR-29b-3p are highlighted due to the high number of their nodes (73 and 67, respectively). Also, genes such as VEGFA appeared regulated by up to 7 different miRNAs (**Supplementary** **Fig. 1a**). Using a p-value<0.01 and an FDR<0.1, we identified 383 genes whose expression was regulated by, at least, two different miRNAs (**Supplementary Table 2**).

The 16 downregulated miRNAs correlated with increased expression of target genes in sarcoma compared to nontumor tissue (**Supplementary** **Fig. 1b**). Of these target genes, at least 56 were transcription factors (TFs), being at least 123 regulated by these TFs (**Supplementary Table 3**). Overall, a minimum of 140 genes from the total of 383, appeared interconnected. Gene Ontology analysis revealed multiple terms related to development, metabolism, and cell motility, associated to increased metastatic capacity of tumor cells, which is related to low 5-year survival rate (**Supplementary** **Fig. 1c**). Therefore, we sought to identify new potential drugs targeting genes related to the 16 downregulated miRNAs. We entered the 383 genes into the DGIdb database, and the resulting list of potential targets was narrowed down to 100 genes and 2071 possible drugs (**Supplementary** **Table 4)**.

To choose possible treatments, we used the GSE21050 sarcoma dataset, containing gene expression, metastases and survival data. miRNA downregulation commonly causes gene upregulation^1^, so we searched for genes from the selected list of 100 genes that were highly expressed in patients with a survival time of less than 5 years. Applying the *Apriori* algorithm, we identified numerous association rules between different genes (**Supplementary Table 5)**. Notably, “metastasis” was the most frequent attribute value in the rules, appearing in more than 60% of the association rules, while “no metastasis” did not appear. Therefore, we looked for the most frequent and strongest associations between highly expressed genes and metastasis in patients with a survival time lower than 5 years. The *Apriori* algorithm identified three common genes: *AKT3,* *CREB1,* and *PAK2* (**Supplementary** **Fig. 2, Supplementary Table 5**), selecting drugs correlating with these genes as biomarkers.

**MATERIALS AND METHODS**

**Cell lines and tumor samples**

Throughout this work, we used different sarcoma cell lines and primary tumors. IBIS-DD, IBIS-DA, AA, AX, AW, BC, BD, BI, BG, and CE lines were previously characterized^2-6^ and used in this study (**Supplementary Table 6**). IMR90, WI-38, A673, HT-1080, Saos-2, and SW872 are commercial cell lines. All cells were maintained as a subconfluent monolayer in different media. IMR90 and WI38 cells were maintained in DMEM (Sigma) supplemented with 10% FBS. A673, HT-1080, Saos2, SW872 and AX cells were maintained in DMEM (Sigma) supplemented with 10% FBS, penicillin/streptomycin antibiotics (Sigma) and Fungizone (Amphotericin B, Sigma). IBIS-DD, IBIS-DA, AA, AW, BC, BD, BI, BG, and CE cells were maintained in F-10 medium (Sigma) supplemented with 10% FBS, penicillin/streptomycin antibiotics (Sigma) and Fungizone (Amphotericin B, Sigma). All cell lines were authenticated and regularly tested for mycoplasma. Each cell line was cultured at 37 °C and 95% humidity in 5% CO_2_ under normoxic conditions.

Tumor and nontumor tissues used for miRNA expression analysis and for PDX generation (**Supplementary Table 7**), as well as muscle samples for WB were obtained via surgical resection performed at Virgen del Rocio Hospital (Seville, Spain). All patients provided written informed consent according to a protocol approved by the local ethics committee (CEI 2013/PI002). S23 and S25 PDX models were derived from undifferentiated pleomorphic sarcoma and were previously described^4,5^. The animal experiments were performed according to the European Guidelines for Laboratory Animal Care. This study was approved by the IBIS Institutional Animal Care and Use Committee.

**PCR array and sample analysis**

Total RNA, both from cells and tissue samples, was extracted with the RNeasy Kit followed by DNase I treatment. PCR assays were performed using the miHS-102Z miScript miRNA PCR Array Human Cancer Pathway Finder (QIAGEN) according to the manufacturer’s instructions. Reverse transcription was performed from 250 ng of total RNA using the miScript II RT kit for cDNA synthesis (QIAGEN). Quantitative real-time PCR was performed (Applied Biosystems® 7900 HT) with an initial activation step at 95 °C for 15 seconds and 40 cycles at 94 °C for 15 seconds, 55 °C for 30 seconds and 70 °C for 30 seconds.

Normalization and data analyses were performed according to the manufacturer’s instructions using their web-based software package. Normalization was carried out using the average of six housekeeping miRNAs: *SNORD61*, *SNORD68*, *SNORD72*, *SNORD95*, *SNORD96A*, and *RNU6-6P*. The expression of the different miRNAs was compared, with the expression of each miRNA in each sarcoma line normalized to the expression in the IMR90 line.

**Bioinformatics**

To study the possible connection between changes in miRNA levels and survival, we used the downregulated miRNAs in OncomiR^7^, choosing a percentile cutoff of 40% for sarcoma and 35% for uterine carcinosarcoma (UCS). To identify relationships between downregulated miRNAs and genes/proteins, we used the MIENTURNET resource^8^. By introducing the 16 miRNAs downregulated in sarcoma into this resource, we obtained a miRNA‒target enrichment, using the miRTarBase tab. From the resulting list, we kept genes with a p value<0.05 and an FDR<0.1 for subsequent analysis, resulting in a list of 383 genes. To further narrow the list to only include genes that have been linked to drugs, we used the Drug Gene Interaction Database resource^9^.

To compare the expression of genes regulated by miRNAs, we used R2 resource (<https://r2.amc.nl>), and selected several normal datasets (GSE7307, GSE3526, GSE9103, GSE3307, GSE1133). For sarcoma datasets, we used Ewing sarcoma (GSE34620, GSE12102, GSE17679, GSE142162), osteosarcoma (GSE14827 and Aqeilan dataset, in R2), and rhabdomyosarcoma (GSE66533) to look for differences in gene expression, which was analyzed using the same probe for each gene in all datasets: *CREB1* (204313_s_at), *PAK2* (208877_at), *AKT3* (212607_at), *HMGCR* (202540_s_at), *ATP13A3* (212297_at), *PTEN* (204053_x_at), *VEGFA* (210512_s_at), *ZNF618* (226592_at), *TGFB1* (203085_s_at), *SHCBP1* (219493_at) and *NFE2L2* (201146_at).

The list of transcriptional regulation pairs is available at TRRUST^10^.

To identify possible treatments, we used the GSE21050 sarcoma database, which combines expression data with patient survival and appearance of metastases. We removed all probes that had low expression levels (log2<5) or showed little variation between patients (log2MAX - log2MIN<1). Using the remaining data, we applied the Apriori algorithm^11^ (available in the arules package of the R software environment) to identify association rules that combine high expression levels of probes with the appearance of metastases and patient survival of less than 5 years. This algorithm requires numerical data to be discretized and requires the setting of parameters such as minimum support and confidence thresholds. The study proposed in this work aims to obtain relationships between probes with high expression levels and patient survival of less than 5 years. Furthermore, identifying whether metastasis was also present in these relationships to detect genes highly related to cancer disease was interesting. To achieve this goal, several steps were taken. First, the numerical variables of the dataset were discretized to convert the continuous variables into categorical variables suitable for the extraction of association rules. On the one hand, the numerical variables associated with probes were discretized into two intervals to categorize the expression levels into low (categorized as 0) or high (categorized as 1) using the equal interval width method. On the other hand, the numerical variable related to patient survival time was discretized using fixed interval boundaries to categorize into survival time less than 5 years (categorized as 0) or greater than or equal to 5 years (categorized as 1). Metastasis has not been discretized because it is already categorized into 0 (absence) or 1 (presence) in the dataset. Next, the Apriori algorithm was applied to the discretized data using a minimum support threshold of 0.05 and a minimum confidence threshold of 1. Note that these measures range into the interval 0 to 1, with our interest focused on obtaining specific association rules with the highest possible reliability. The length range was set from 3 (minimum) to 5 (maximum) to reduce the set of rules to be found by Apriori. In the context of the proposed study, the Apriori algorithm was restricted to obtain association rules by setting the antecedent of the rules with probes categorized as 1 (high expression levels) and consequent of the rules with patient survival time categorized as 0 (less than 5 years). Because our goal was to detect whether metastasis is involved in the most relevant rules with the conditions mentioned above, that metastasis appears with specific categories in the rules was not fixed. Third, all probes appearing in the association rules discovered by Apriori were converted into official gene symbols. Then, we obtained the top of frequent genes joining all genes found in the rules. Furthermore, we also calculated the number of rules that also contain metastasis as an attribute in the rules to quantify its relevance in the set of rules obtained.

**WB and gene transcription analysis**

Protein extracts and mRNA were obtained as previously described^12,13^. To obtain protein extracts from PDX tumors and nontumor tissue samples, the tissues were homogenized using a mortar and liquid nitrogen, resuspended in lysis buffer (Trizma 50 mM, pH 7.4, sucrose 0.25 mM, EDTA 5 mM, Triton 0.5%) supplemented with a complete protease inhibitor cocktail (P8340, Sigma). The cells were then lysed by sonication and centrifuged at 10000 × g for 10 minutes at 4 °C.

To detect changes in gene expression, we used the following probes: *CREB1* (Hs.PT.8.1208595), *PAK2* (Hs.PT.58.2959262), *AKT3* (Hs.PT.58.2121896, and *ACTB* (Hs.PT.39a.22214847) from IDT. Polymerase chain reactions (PCRs) were performed as previously described^12,13^.

For WB, we used CREB1 (sc-377154, Santa Cruz Biotechnologies), PAK2 (Sc-373740, Santa Cruz Biotechnologies) and AKT3 (sc-134254, Santa Cruz Biotechnologies) antibodies. α-Tubulin (T9026, Sigma) and HSP70 (ab45133, Abcam) antibodies were used as controls. Horseradish peroxidase-labeled rabbit anti-mouse (ab97046, Abcam) or goat anti-rabbit (ab97051, Abcam) secondary antibodies were also used.

**IC50 assays**

To determine whether changes in gene expression lead to changes in cell resistance, cells were seeded in a 96-well plate at different densities according to the cell line. In this way, WI38, IMR90, AA, AW, AX, BC, BG and CE cells were seeded at 15000 cells/well, while Saos2 cells were seeded at 10000 cells/well, and A673, SW872 and HT-1080 cells were seeded at 5000 cells/well. All cells were treated with the different drugs 24 hours later. The initial concentrations for individual treatments were 1 mM citalopram, 65 µM cisplatin, 100 µM vemurafenib, or 100 µM PF-03758309, with 1/3 serial dilutions. For double treatments, an additional concentration of 40 µM citalopram or 2 µM cisplatin was used. After 96 hours, the cells were fixed with 4% glutaraldehyde and stained with crystal violet. The IC50 value was calculated using GraphPad Prism software.

**PDX generation and *in vivo* treatments**

PDXs were generated as previously described^4^. Each tissue was divided into two parts. One part was frozen, and the remaining part was cut into small fragments 2–3 mm in diameter and used for subcutaneous implantation into 6-week-old female Foxn1nu athymic nude mice (Harlan Laboratories, The Netherlands).

Experiments were performed using cohorts of six animals for each group. Mice were randomly allocated to the following groups: citalopram, PF-03758309, vemurafenib, cisplatin, citalopram plus PF-03758309, citalopram plus vemurafenib, PF-03758309 plus vemurafenib, citalopram plus cisplatin, PF-03758309 plus cisplatin, vemurafenib plus cisplatin and control (solvent only). Once the tumor reached a size of 20 mm^3^, mice were treated with 100 mg/kg citalopram in water bottles, with 15 mg/kg PF-03758309 5 times a week, with 10 mg/kg vemurafenib 3 times a week, or with 2 mg/kg cisplatin twice a week. All treatments lasted for 3 weeks. Mice were monitored daily for distress signs, and no signs of toxicity were observed. Tumor size was measured using a caliper as previously described^4^. After 25-30 days, the mice were sacrificed, and the tumors were extracted and stored at -80 °C. All animal experiments were conducted according to the experimental protocol approved by the Institutional Animal Care and Use Committee of IBIS and HUVR (0309-N-15).

Supplementary Text

**ABBREVIATIONS**

5-FU, 5-fluorouracil

α-TUB, Tubulin alpha isoform

AKT3, v-akt murine thymoma viral oncogene homolog 3

BRAF^V600E^, B-RAF protooncogene mutated at V600E, serine/threonine kinase

CREB1, AMP response element-binding protein 1

CSCs, Cancer Stem Cells

ECM, Extracellular Matrix

EMT, Epithelial to Mesenchymal Transition

FBS, Fetal bovine serum

FDR, False Discovery Rate

GO, Gene Ontology

HIV, Human immunodeficiency virus

HSP70, Heat shock protein 70 KD

IC50, Inhibitory concentration at 50% reduction

miRNA, microRNA

PAK2, p21 protein-activated kinase 2

PDX, Patient derived xenograft

qPCR, Quantitative PCR

qRT‒PCR, Reversetranscriptase quantitative PCR

TFs, transcription factors

WB, Western blot

Figure. S1.


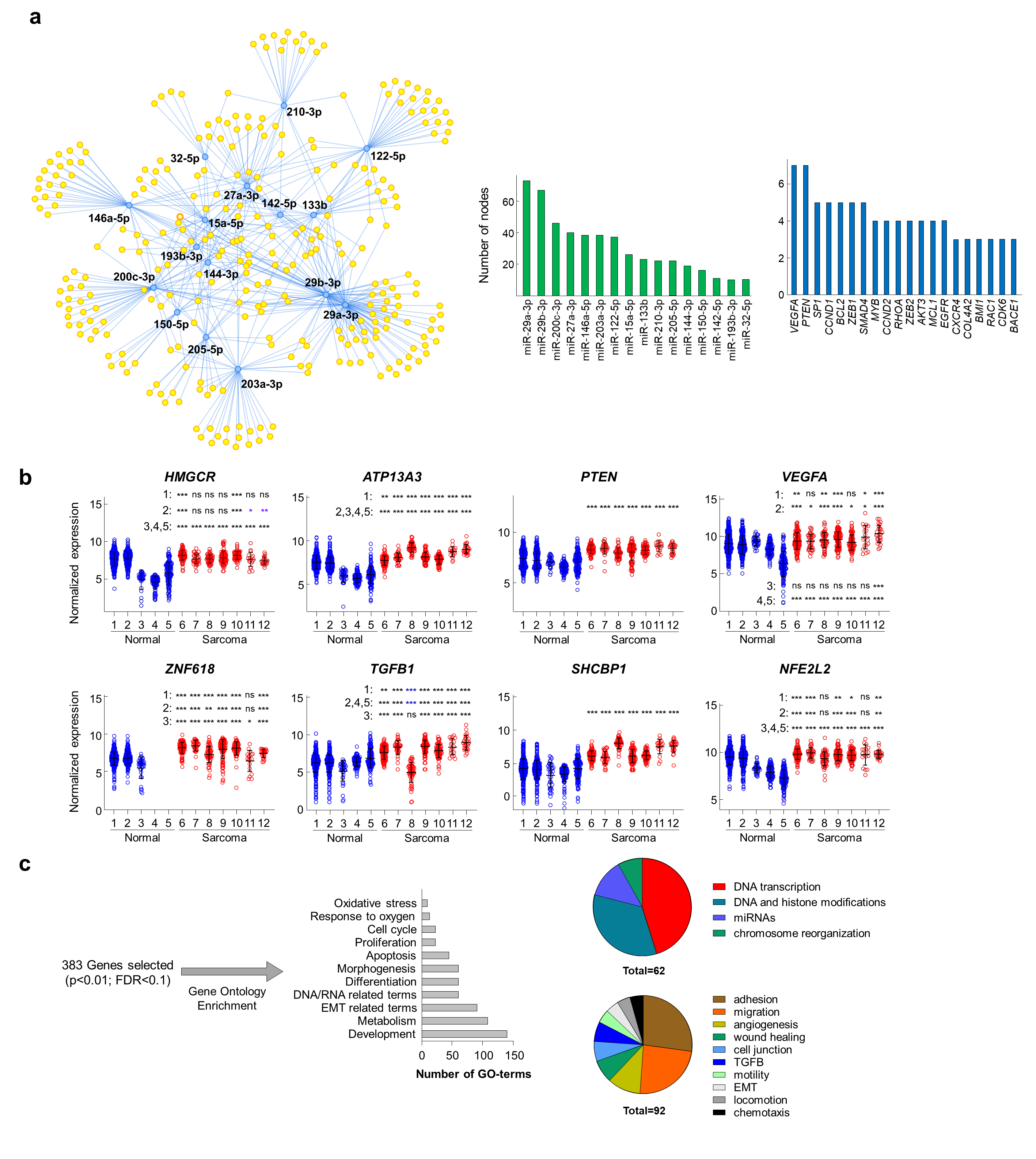


**Supplementary Figure 1. a)** The relationship between downregulated miRNAs in sarcoma and genes regulated by these miRNAs was determined using the MIENTURNET resource. A large interconnection between miRNAs through the regulation of common genes was observed. For example, miR-29a-3p has 73 nodes, representing its relationship with 73 different genes, while at the gene level, VEGFA and PTEN are regulated by 7 miRNAs from the list obtained. **b)** Modification in the expression of some of the genes regulated by downregulated miRNAs in sarcomas. (1: GSE7307, 2: GSE3526, 3: GSE9103, 4: GSE3307, 5: GSE1133 6: GSE34620, 7: GSE13102, 8: GSE66533, 9: GSE17679, 10: GSE142162, 11: Aqeilan dataset (R2), 12: GSE14827). In blue, samples from normal datasets; in red, samples from sarcoma datasets. Black asterisks indicate gene upregulation, blue asterisks indicate gene downregulation, and ns indicates nonsignificant. *: p value<0.05, **: p value<0.01; ***: p value<0.001. **c)** Genes with p value<0.01 and FDR<0.1 were used to perform a Gene Ontology analysis, finding, among the most abundant terms, those related to DNA/RNA or to epithelial-mesenchymal transition.

Supplementary Figure 2.


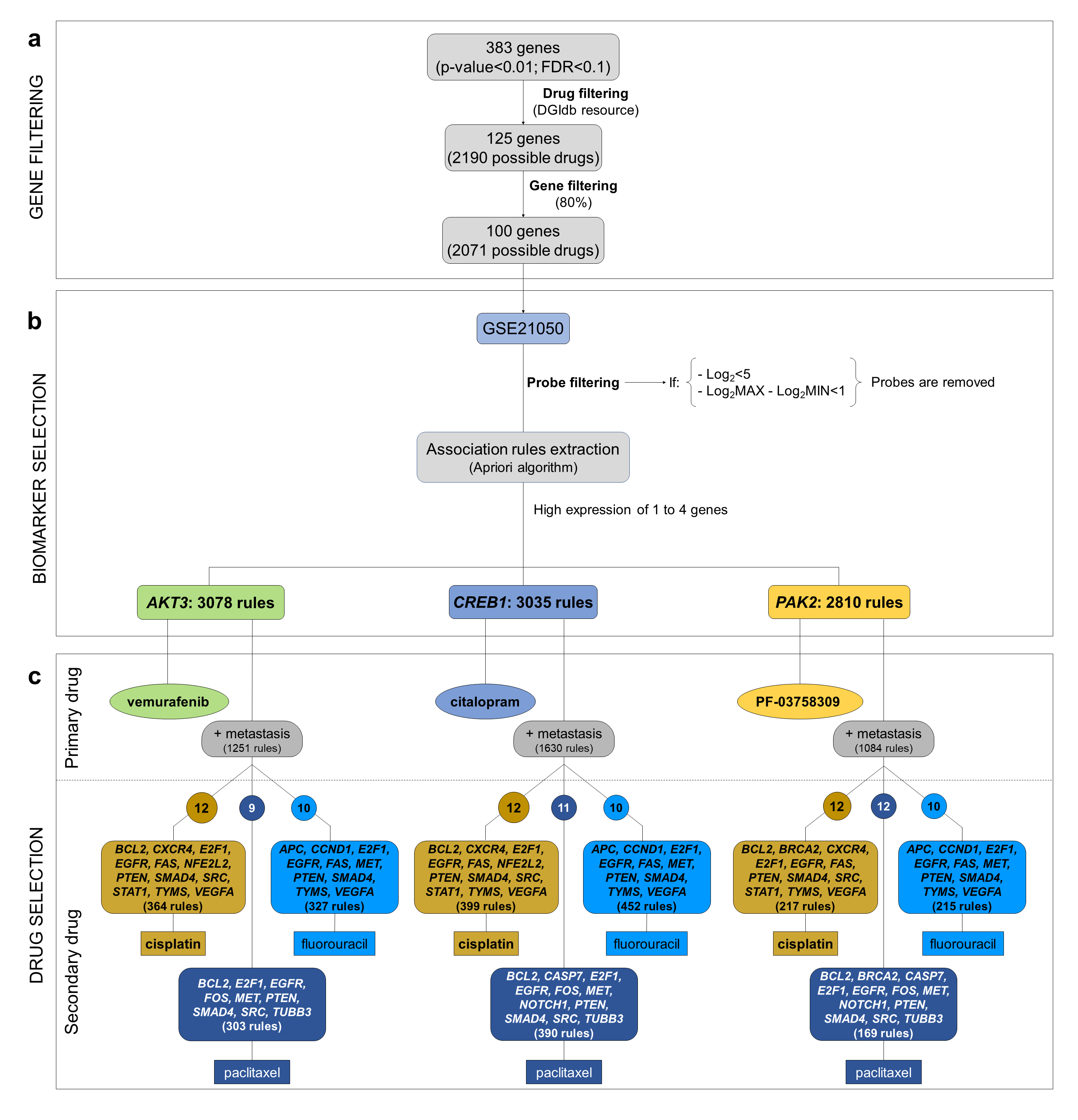


**Supplementary Figure 2.** Flow chart of the strategy followed for the selection of drugs to treat sarcomas, starting from the list of 383 genes whose expression was regulated by, at least, two different miRNAs. **a)** Gene filtering to select only those with associated drugs, according to the DGIdb resource. We kept 80% of the genes with the highest significant values from the list of 125 genes to continue with the analysis. **b)** The GSE21050 dataset was used to find gene association rules using the a priori algorithm, after discarding probes with small expression (log_2_<5) or no changes (log_2_MAX-log_2_MIN<1) between samples. By doing this, we obtained *AKT3*, *CREB1* and *PAK2* as the genes with the highest number of gene association rules. **c)** Vemurafenib, citalopram and PF-03758309 were selected as primary drugs, based on gene-drug associations, and cisplatin was selected as a secondary drug, over fluorouracil or paclitaxel, due to the higher number of genes associated with metastasis and *AKT3*, *CREB1* or *PAK2*.

**Supplementary Figure 3.**

**
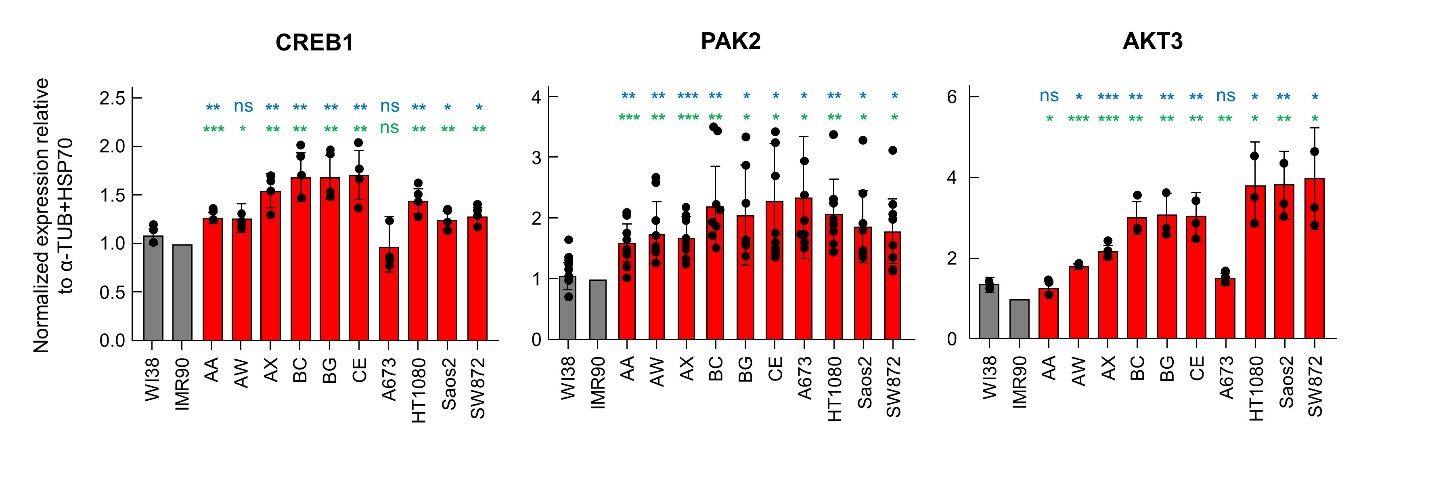
**

**Supplementary Figure 3.** The normalized expression of the three sarcoma biomarkers CREB1, PAK2 and AKT3 in the sarcoma cell line panel used in this study is shown. Quantitative data was extracted from **Figure 1e** and normalized to tubulin and HSP70 to ensure comparable levels among all cell lines.

**Supplementary Figure 4.**


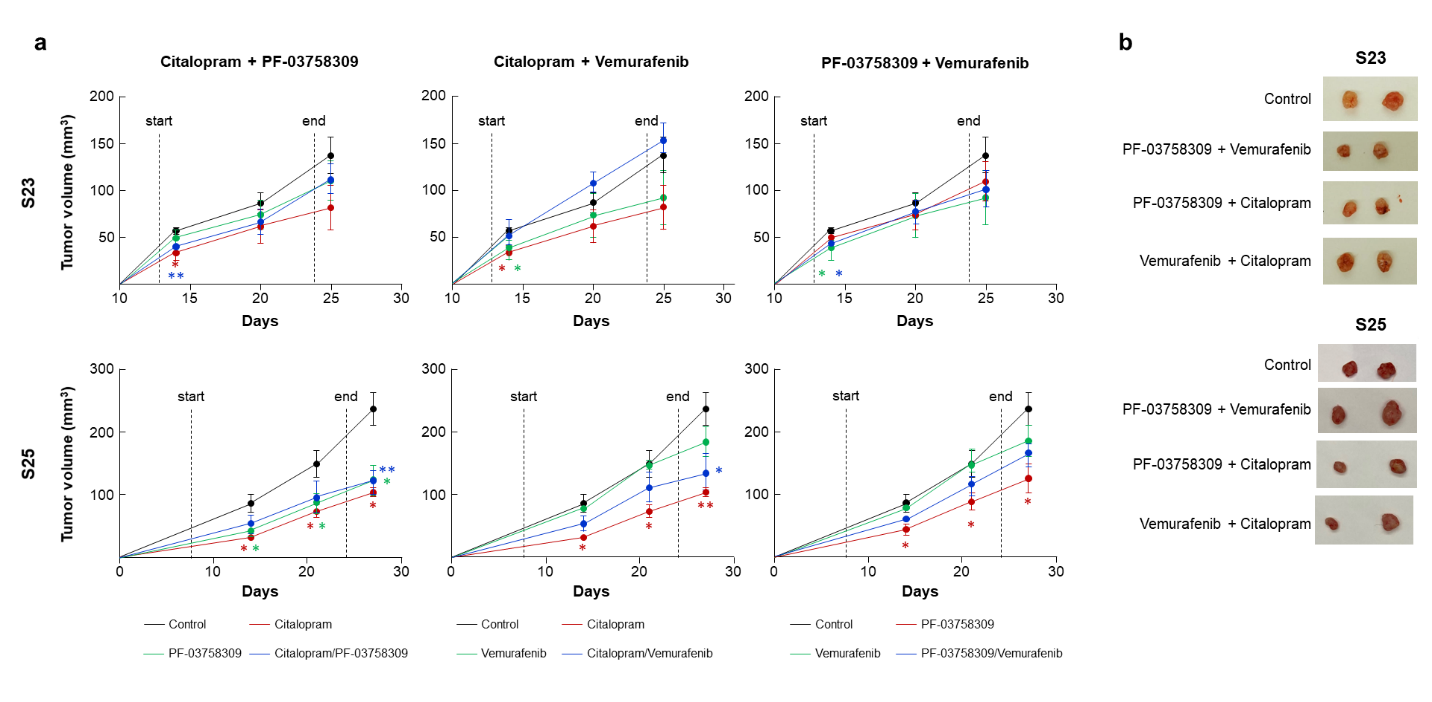


**Supplementary Figure 4. Effect of other individual or combined treatments on sarcoma tumors in vivo.** **a)** PDXs generated in mice were treated with citalopram, PF-03758309, vemuranib, cisplatin, or a combination of these, and most treatments resulted in a reduction in tumor volume. Start and End refer to the extension of each treatment. **b)** PDX tumors were surgically extracted at the end, showing final volume due to treatments with citalopram, citalopram, PF-03858309 or vemurafenib (or among them).

Supplementary Table 1.

| **Supplementary Table 1.** List of dysregulated miRNAs in sarcoma cell lines and sarcoma tumors. | | | |
| --- | --- | --- | --- |
| Cell lines vs IMR90 | | Sarcoma vs normal tissue | |
| down | up | down | up |
| hsa-miR-150-5p | hsa-miR-34c-5p | hsa-miR-206 | hsa-miR-138-5p |
| hsa-miR-200c-3p | hsa-miR-214-3p | hsa-miR-1-3p | hsa-miR-124-3p |
| hsa-miR-10a-5p | hsa-miR-143-3p | hsa-miR-205-5p |  |
| hsa-miR-29b-3p | hsa-miR-19a-3p | hsa-miR-144-3p |  |
| hsa-miR-127-5p | hsa-miR-96-5p | hsa-miR-133b |  |
| hsa-miR-203a-3p | hsa-miR-100-5p | hsa-miR-203a-3p |  |
| hsa-miR-122-5p | hsa-miR-222-3p | hsa-miR-122-5p |  |
| hsa-miR-32-5p | hsa-miR-132-3p | hsa-miR-378a-3p |  |
| hsa-miR-130a-3p | hsa-miR-1-3p | hsa-miR-23b-3p |  |
| hsa-miR-193b-3p | hsa-miR-148b-3p | hsa-miR-27b-3p |  |
| hsa-miR-215-5p | hsa-miR-98-5p | hsa-miR-200c-3p |  |
| hsa-miR-144-3p | hsa-miR-7-5p | hsa-miR-29b-3p |  |
| hsa-miR-372-3p | hsa-miR-148a-3p | hsa-miR-150-5p |  |
| hsa-miR-34a-5p | hsa-miR-206 | hsa-miR-143-3p |  |
| hsa-miR-149-5p | hsa-miR-138-5p | hsa-miR-193b-3p |  |
| hsa-miR-134-5p | hsa-miR-183-5p | hsa-miR-27a-3p |  |
| hsa-miR-373-3p | hsa-miR-27b-3p | hsa-miR-128-3p |  |
| hsa-miR-133b | hsa-let-7b-5p | hsa-miR-140-5p |  |
| hsa-miR-181c-5p | hsa-miR-378a-3p | hsa-miR-29a-3p |  |
| hsa-miR-181a-5p | hsa-miR-146b-5p | hsa-let-7f-5p |  |
| hsa-miR-29a-3p | hsa-miR-155-5p | hsa-let-7a-5p |  |
| hsa-miR-301a-3p | hsa-miR-218-5p | hsa-let-7c-5p |  |
| hsa-miR-142-5p | hsa-miR-23b-3p | hsa-miR-126-3p |  |
| hsa-miR-124-3p | hsa-miR-191-5p | hsa-miR-98-5p |  |
| hsa-miR-146a-5p | hsa-let-7a-5p | hsa-miR-20b-5p |  |
| hsa-miR-210-3p | hsa-miR-125a-5p | hsa-miR-20a-5p |  |
| hsa-miR-184 | hsa-miR-126-3p | hsa-miR-15a-5p |  |
| hsa-miR-205-5p | hsa-let-7e-5p | hsa-miR-146a-5p |  |
| hsa-miR-181d-5p | hsa-miR-92a-3p | hsa-let-7b-5p |  |
| hsa-miR-27a-3p | hsa-miR-30c-5p | hsa-miR-30c-5p |  |
| hsa-miR-15a-5p | hsa-miR-125b-5p | hsa-let-7g-5p |  |
| hsa-miR-181b-5p | hsa-miR-21-5p | hsa-miR-16-5p |  |
|  | hsa-miR-18a-5p | hsa-let-7d-5p |  |
|  | hsa-let-7f-5p | hsa-miR-10b-5p |  |
|  | hsa-let-7d-5p | hsa-miR-17-5p |  |
|  | hsa-let-7g-5p | hsa-miR-142-5p |  |
|  | hsa-miR-20b-5p | hsa-miR-210-3p |  |
|  | hsa-miR-20a-5p | hsa-miR-96-5p |  |
|  | hsa-miR-128-3p | hsa-miR-196a-5p |  |
|  | hsa-let-7i-5p | hsa-let-7e-5p |  |
|  | hsa-miR-17-5p | hsa-miR-18a-5p |  |
|  | hsa-miR-140-5p | hsa-miR-148b-3p |  |
|  | hsa-miR-25-3p | hsa-miR-19a-3p |  |
|  | hsa-miR-10b-5p | hsa-miR-214-3p |  |
|  | hsa-miR-16-5p | hsa-miR-32-5p |  |
|  | hsa-miR-15b-5p | hsa-miR-148a-3p |  |
|  | hsa-miR-9-5p | hsa-let-7i-5p |  |
|  | hsa-let-7c-5p | hsa-miR-218-5p |  |
|  | hsa-miR-196a-5p | hsa-miR-183-5p |  |

Supplementary Tables S2-S5.

See Excel file “supplementary tables”

**Supplementary Table 6.**

| **Supplementary Table 6**. List of sarcoma cell lines used in this study | | | |
| --- | --- | --- | --- |
| Cell line | Tumor of Origin | | Phenotype |
| IBIS-DD | Myxoid Fibrosarcoma | | NA |
| IBIS-DA | MPNST | | NA |
| AA | Leiomyosarcoma | | Epithelial |
| AX | Myxoid Liposarcoma | | Epithelial |
| AW | Myxoid Liposarcoma | | Fibroblastic |
| BC | MPNST | | Epithelial |
| BD | Ewing Sarcoma | | NA |
| BI | GIST | | NA |
| BG | Myxoid Fibrosarcoma | | Fibroblastic |
| CE | Rhabdomyosarcoma | | Fibroblastic |
| A673 | Ewing Sarcoma | | Fibroblastic |
| HT-1080 | Fibrosarcoma | | Epithelial |
| Saos-2 | Osteosarcoma | | Epithelial |
| SW872 | Liposarcoma | | Fibroblastic |
| NA: Not Available | |  |  |

| **Supplementary Table 7.** Characteristics of the sarcoma tumors used in this study. | | | | | |
| --- | --- | --- | --- | --- | --- |
| Sample | HISTOLOGICAL DIAGNOSIS | AGE | SEX | METASTATIC (YES/NO) | PREVIOUS TREATMENTS |
| **S06T** | Soft tissues, right thigh (exeresis) - Muscle-aponeurotic fibromatosis close to the resection margins. | 56 | F | Local recurrence | NO |
| **S07T** | Pelvis, retroperitoneum (biopsy and resection) - Retroperitoneal recurrence of undiferentiated sarcoma. - Infiltration by undiferentiated sarcoma of the left pelvis, supravesical, meso and left pelvic wall. | 52 | F | YES | NO |
| **S11T** | Soft tissue, knee (en bloc resection) - High grade myxofibrosarcoma of 11 cm that does not reach the resection margins and infiltrates into the middle dermis, respecting the epidermis. - Fibroadipose fragment with fibrosis, without evidence of infiltration due to neoplasm (peritumoral lesion). Comment: Grade 3 (6) of the FNCLCC. The neoplasia only shows expression for vimentin, with the rest of the immunohistochemical markers being negative (AML, PAN-CK, desmin, MDM2, melan A, myogenin and S100). High proliferative index with a Ki67 of 40%. | 66 | M | YES | NO |
| **S12T** | Bone. Left femur (post-chemotherapy resection):  - High grade conventional osteosarcoma:  · Tumor size: 8.5 cm.  · Degree of histological response to chemotherapy IIB (28% viable tumor)  - Adjacent soft tissue infiltration  · Distal resection margin: free of neoplasia  · Circumferential resection margin: free of neoplasia  COMMENT: The neoformation shows extensive areas of chondroblastic differentiation. | 36 | F | NO | Neoadjuvant |
| **S13T** | Retroperitoneum (resection), recurrence:  - Differentiated liposarcoma:  · Tumor size: 28 cm.  · Histological grade 2 of the FNCLCC: 3+1+1  · Surgical resection margin affected by neoplasm. | 54 | F | YES | Radiotherapy after primary surgery. |
| **S14T** | Soft parts - gluteal region (exeresis);  - Malignant tumor of the peripheral nerve sheath, grade 2, in contact with the resection margins.  - Histological grade (according to FNCLCC/NCI): - Tumor grade: 2; Mitotic index: 2; Necrosis: 1; Vascular invasion has not been demonstrated.  Comment: An immunohistochemical study has been carried out that has shown expression of S100 in tumor cells, with negativity for CD34, MDM2, desmin, EMA and Bcl2. Other markers (smooth muscle actin, HMB45 and melan A) were negative, performed in a previous biopsy (11B20070). The proliferation rate is high 50-60%). | 66 | M | NO | NO |
| **S15T** | Soft parts- ankle (resection). - Mixo-inflammatory fibroblastic sarcoma, which contacts the resection margin. - Histological grade: 1: Tumor differentiation grade: 1; Mitotic index: 1;necrosis: 0 | 32 | F | NO | NO |
| **S16T** | Soft tissue of the right thigh, femur bone (resection) - myxofibrosarcoma grade 2 of the FNCLCC of 8 cm that respects the resection margins, approximate to 5 mm. - cortical bone fragments of the femur without evidence of infiltration by myxofibrosarcoma. Comment: It is a lesion with expansive margins, made up of a proliferation of spindle cells with moderate atypia and large myxoid areas arranged in nodules; however, dense cellular areas of severe atypia with moderate necrosis and high mitotic count (15 mitoses per 10AGC) can be seen. | 83 | F | YES | NO |
| **S17T** | Soft parts (exeresis): - Soft tissue infiltration by grade 1 chondrosarcoma which respects resection planes. - Skin and subcutaneous cell tissue without relevant alterations. | 44 | M | YES | NO |
| **S18T** | Retroperitoneum (exeresis): - Totally necrosed tumor not evaluable. NOTE: a wide inclusion of the lesion has been made, observing totally necrotic tumor tissue that does not allow assessing the tumor lineage. Only preserved cells are observed in the periphery of the tumor that correspond to intact adrenal cortex, which suggests that the tumor arises from this gland, or else in its periphery; however, the absence of valuable morphological data does not allow us to confirm the exact origin. Tumor necrosis also precludes an immunohistochemical study to assess the lineage of the neoplasia. | 70 | M | NO | NO |
| **S19T** | Bone- humerus (biopsy):  - Low grade cartilaginous neoplasm compatible with enchondroma.  Comment: It is not possible to make the histological distinction between enchondroma and low-grade or grade 1 chondrosarcoma. | 45 | F | NO | NO |
| **S20T** | Bone. Right distal femur (resection):  - parostal osteosarcoma:  · tumor size: 6 cm.  · Extension to adjacent soft tissues.  · Lateral resection margins free of neoplasm  · Circumferential resection margin free of neoplasia (less than 1 cm.) | 37 | F | NO | NO |
| **S21T** | Pleura and lung (VATS and resection-biopsy):  - Monophasic synovial sarcoma  Comment: immunophenotype of the neoplasm (H-607-12): expression of Bcl2 and CD99 in the neoplasm, focal in CKAE1/AE3 tubular structures, being negative for smooth muscle actin (AML), S100, desmin, CD34. Medium proliferative index (ki 67: expression between 20-30%). I request a molecular study for SYT translocation.  - Histological evaluation of sarcomas according to FNCLCC: grade 2: degree of differentiation (2); mitotic index (2); between 10 -19 mitoses per 10 CGA; Necrosis (0), absent. | 20 | F | YES | NO |
| **S23T** | Soft tissue armpit, axillary lymph node (en bloc resection) Undiferentiated pleomorphic sarcoma in contact with resection margins. Comment: Pleomorphic neoplasm similar to the previous biopsy (12B1638) with large areas of necrosis and immunohistochemical profile with expression of vimentin and focal for CD138 with negativity for the rest of the markers (AML, S100, CD4, CD68, CKAE1/AE3, among others). It is not possible to prove that the lesion is in a lymph node. The axillary nodes show reactive lymphadenitis with no evidence of involvement by the neoplasia. | 26 | M | NO | Neoadjuvant |
| **S24T** | Uterus and adnexa (total hysterectomy and double adnexectomy)  - adenosarcoma of 20 cm with overgrowth of the stroma of the right ovary.  - atrophic endometrium.  - chronic cervicitis with squamous metaplasia.  - luteinized fibrotecoma of the left ovary.  Comment: Given the size of the tumor and its imprecise limits, it is not possible to ensure staging; however, we consider it to be limited to the right ovary, which would be a FIGO IB stage. | 64 | F | YES | NO |
| **S25T** | Soft parts. Back of leg (resection):  - Undiferentiated pleomorphic sarcoma: · tumor size: 11 cm.  · histological grade 3 of the FNCLCC: differentiation (3); mitosis (3); necrosis (2)  · resection margins free of neoplasma (1mm). · no evidence of vascular permeation. · tumor stage (tnm): pt2a, nx, mx. | 78 | M | NO | NO |

**SUPPLEMENTARY REFERENCES**

1 Ha, M. & Kim, V. N. Regulation of microRNA biogenesis. *Nature Reviews Molecular Cell Biology* **15**, 509-524, (2014).

2 Moneo, V. *et al.* Extreme sensitivity to Yondelis (Trabectedin, ET-743) in low passaged sarcoma cell lines correlates with mutated p53. *J Cell Biochem* **100**, 339-348, (2007).

3 Moneo, V. *et al.* Levels of p27(kip1) determine Aplidin sensitivity. *Mol Cancer Ther* **6**, 1310-1316, (2007).

4 Perez, M. *et al.* Sarcoma stratification by combined pH2AX and MAP17 (PDZK1IP1) levels for a better outcome on doxorubicin plus olaparib treatment. *Signal Transduct Target Ther* **5**, 195 (2020).

5 Perez, M., Muñoz-Galván, S., Jiménez-García, M. P., Marín, J. J. & Carnero, A. Efficacy of CDK4 inhibition against sarcomas depends on their levels of CDK4 and p16ink4 mRNA. *Oncotarget* **6**, 40557-40574, (2015).

6 Perez, M. *et al.* Efficacy of bortezomib in sarcomas with high levels of MAP17 (PDZK1IP1). *Oncotarget* **7**, 67033-67046, (2016).

7 Wong, N. W., Chen, Y., Chen, S. & Wang, X. OncomiR: an online resource for exploring pan-cancer microRNA dysregulation. *Bioinformatics* **34**, 713-715, (2017).

8 Licursi, V., Conte, F., Fiscon, G. & Paci, P. MIENTURNET: an interactive web tool for microRNA-target enrichment and network-based analysis. *BMC Bioinformatics* **20**, 545, (2019).

9 Freshour, S. L. *et al.* Integration of the Drug–Gene Interaction Database (DGIdb 4.0) with open crowdsource efforts. *Nucleic Acids Research* **49**, D1144-D1151, (2020).

10 Han, H. *et al.* TRRUST v2: an expanded reference database of human and mouse transcriptional regulatory interactions. *Nucleic Acids Res* **46**, D380-d386, (2018).

11 Hahsler, M., Grün, B. & Hornik, K. arules - A Computational Environment for Mining Association Rules and Frequent Item Sets. *Journal of Statistical Software* **14**, 1 - 25, (2005).

12 Garcia-Heredia, J. M., Lucena-Cacace, A., Verdugo-Sivianes, E. M., Pérez, M. & Carnero, A. The Cargo Protein MAP17 (PDZK1IP1) Regulates the Cancer Stem Cell Pool Activating the Notch Pathway by Abducting NUMB. *Clinical Cancer Research* **23**, 3871-3883, (2017).

13 García-Heredia, J. M., Verdugo Sivianes, E. M., Lucena-Cacace, A., Molina-Pinelo, S. & Carnero, A. Numb-like (NumbL) downregulation increases tumorigenicity, cancer stem cell-like properties and resistance to chemotherapy. *Oncotarget* **7**, 63611-63628, (2016).

Original images for WB (Figures 1e and 1g)


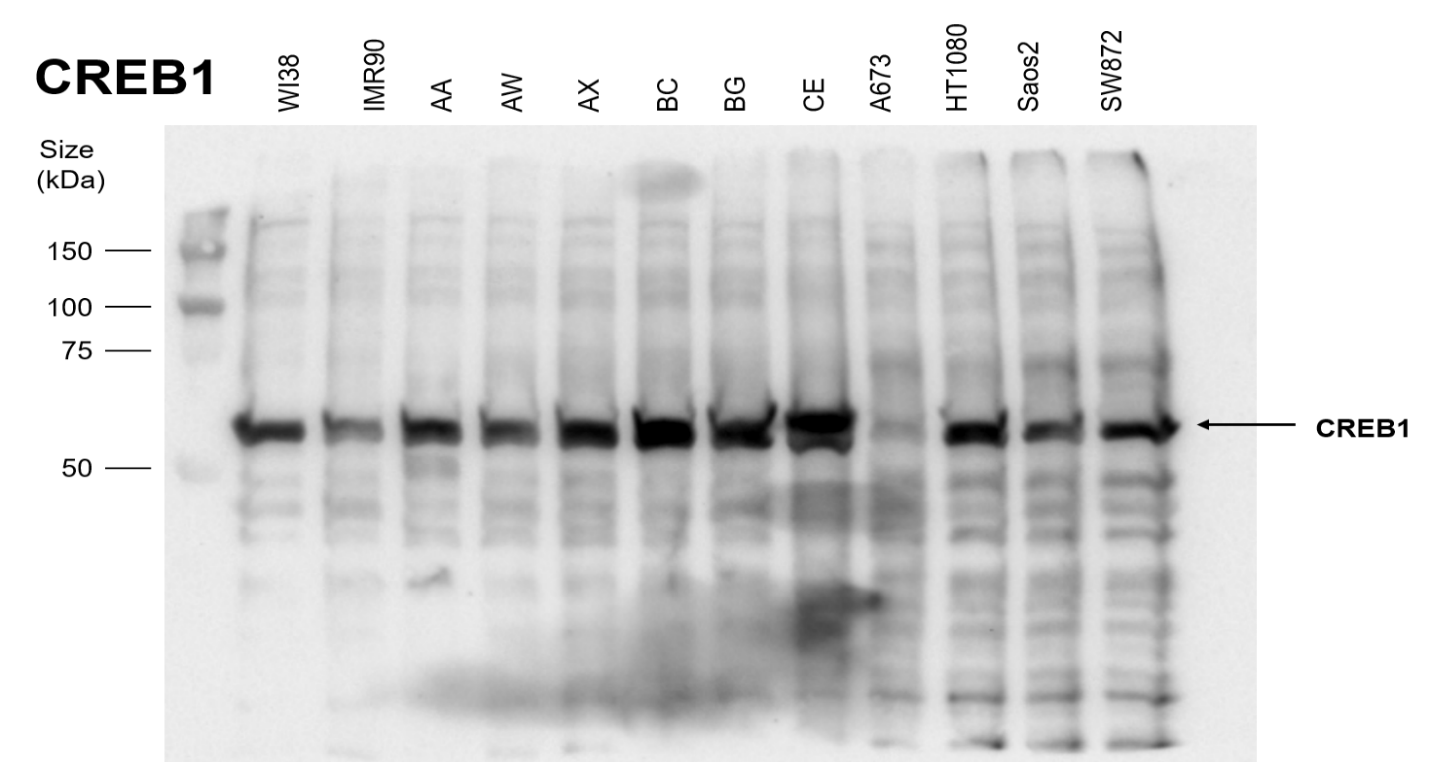


Original images for WB (Figures 1e and 1g)


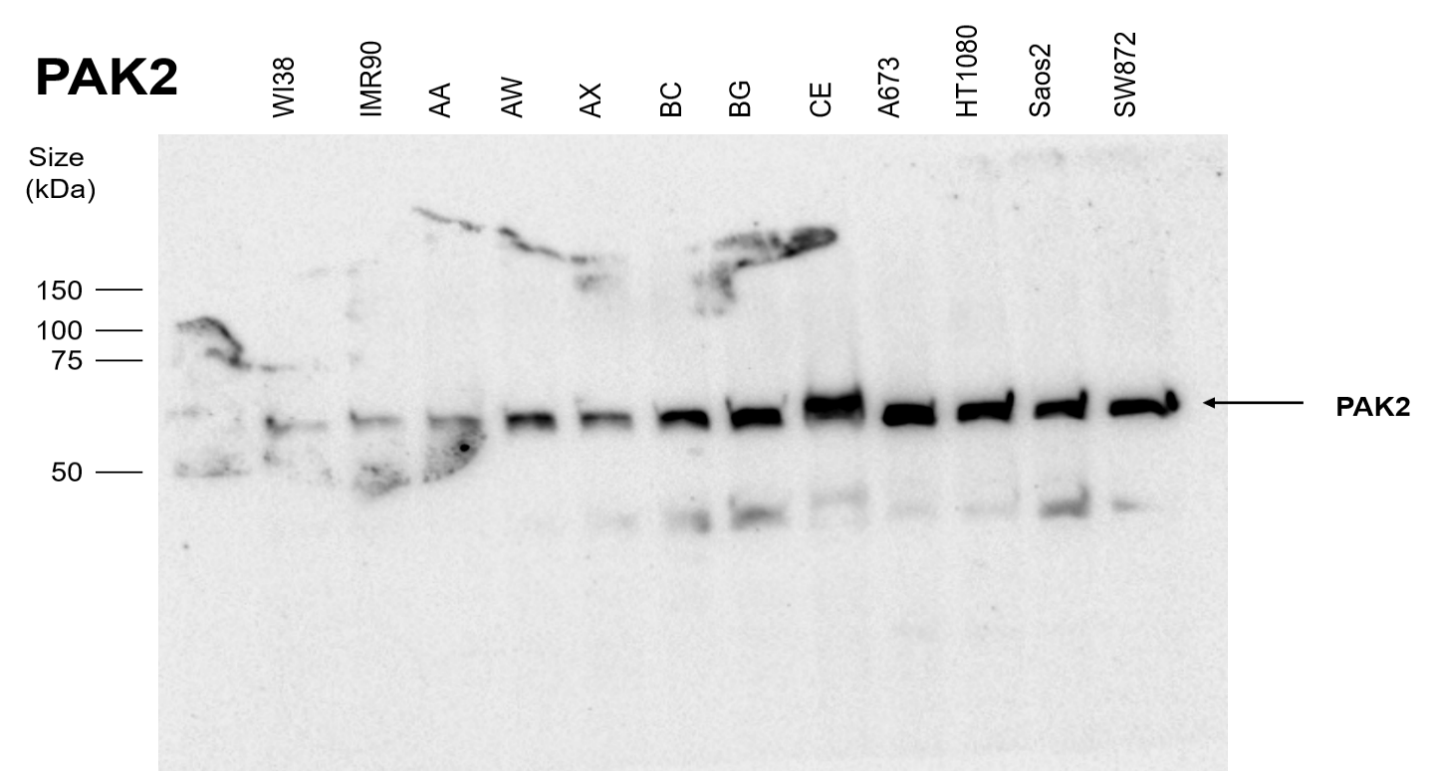


Original images for WB (Figures 1e and 1g)


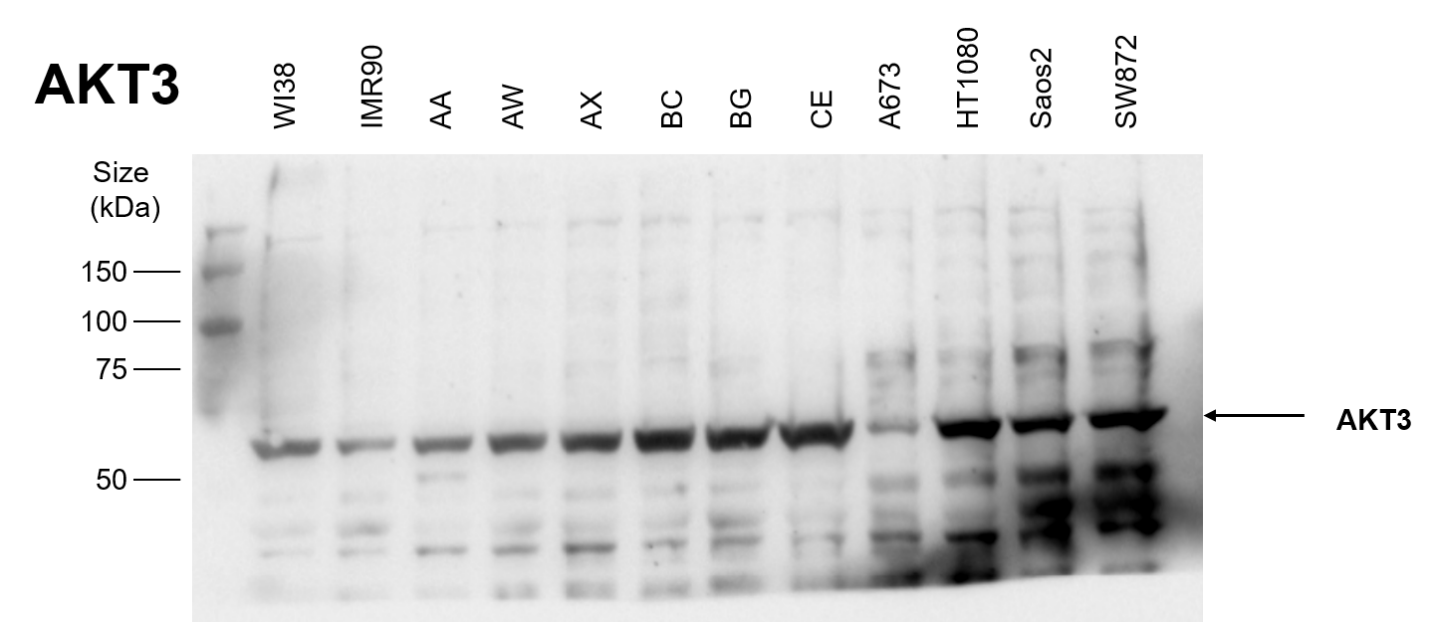


Original images for WB (Figures 1e and 1g)


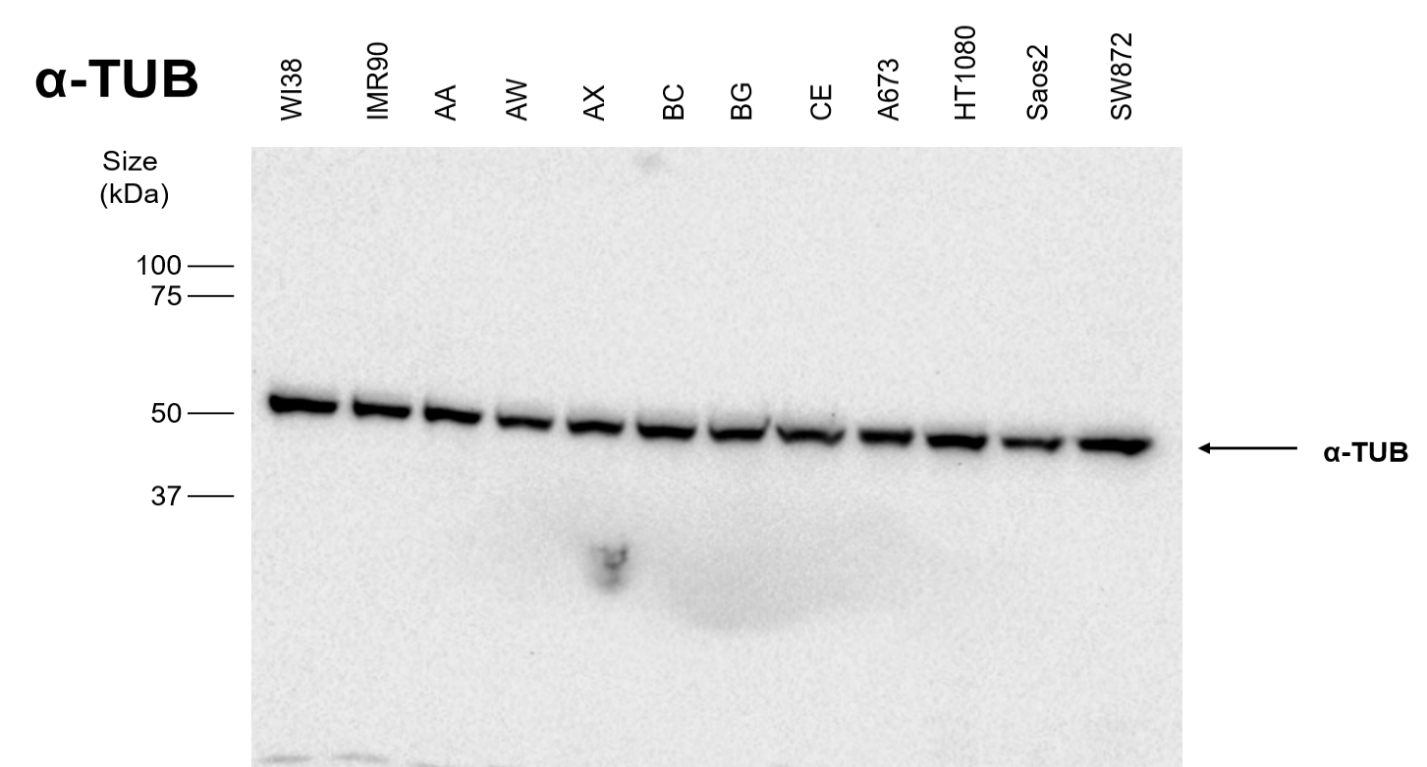


Original images for WB (Figures 1e and 1g)


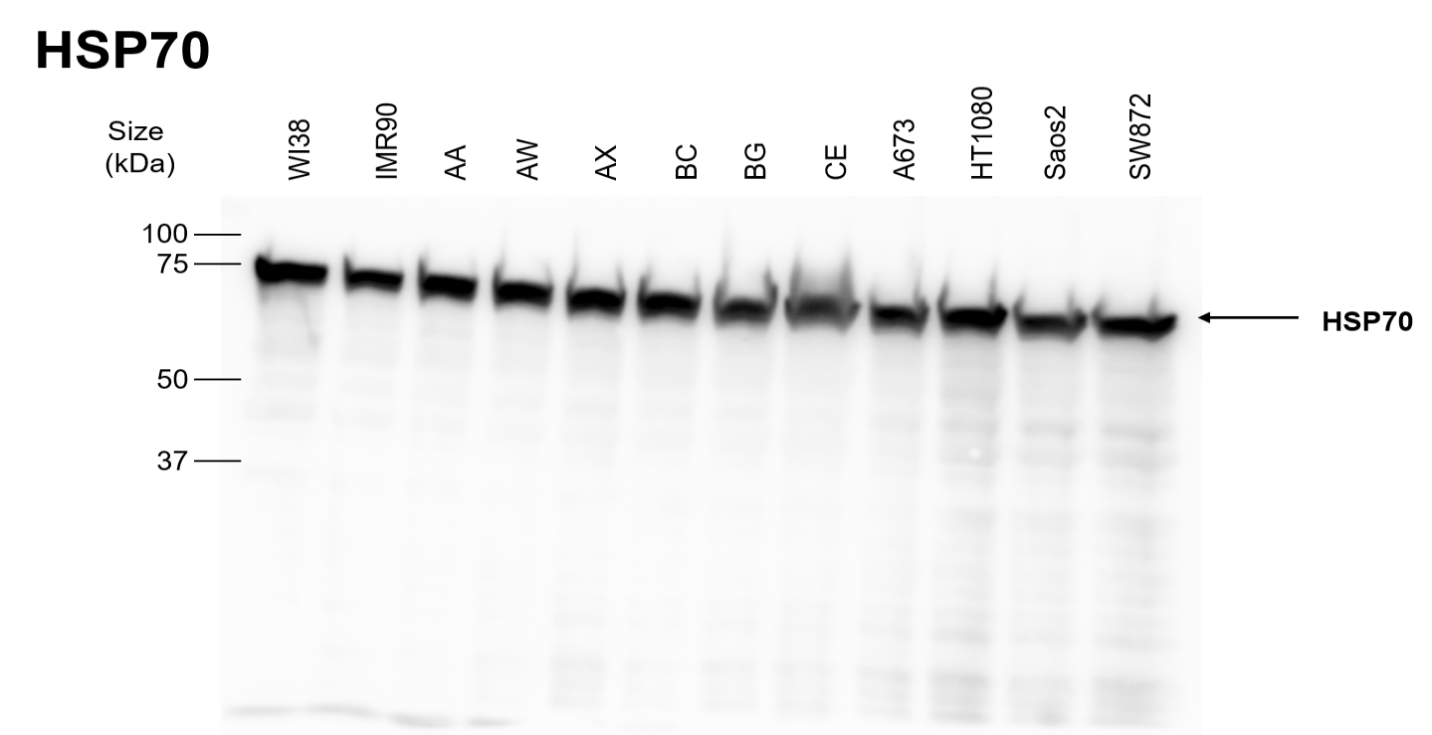


Original images for WB (Figures 1e and 1g)


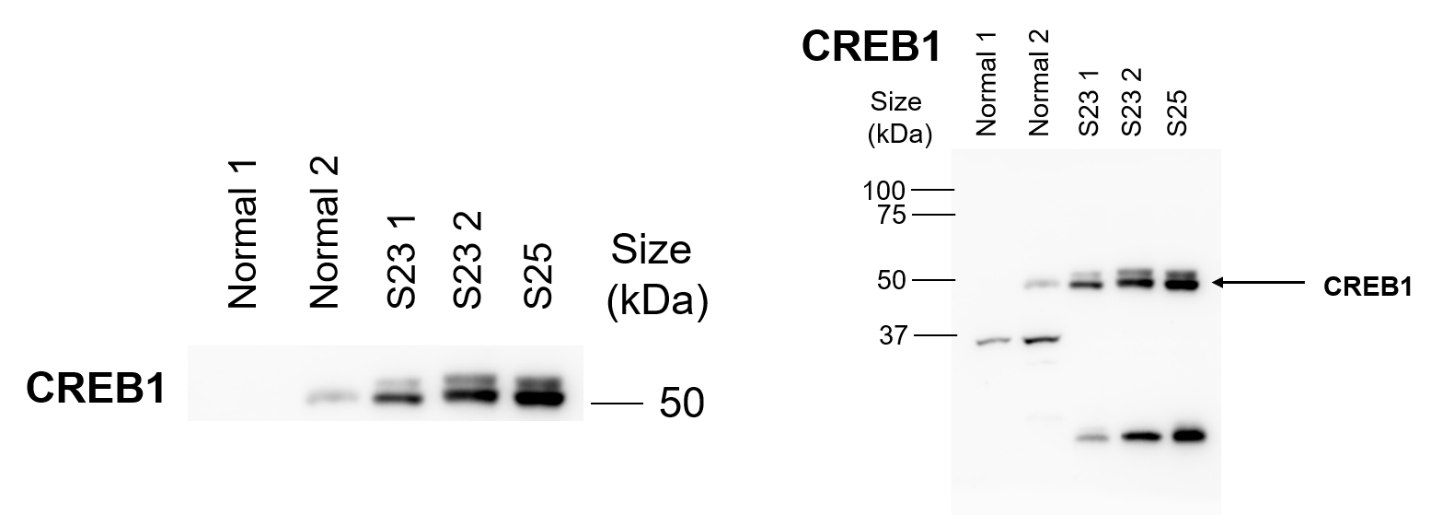


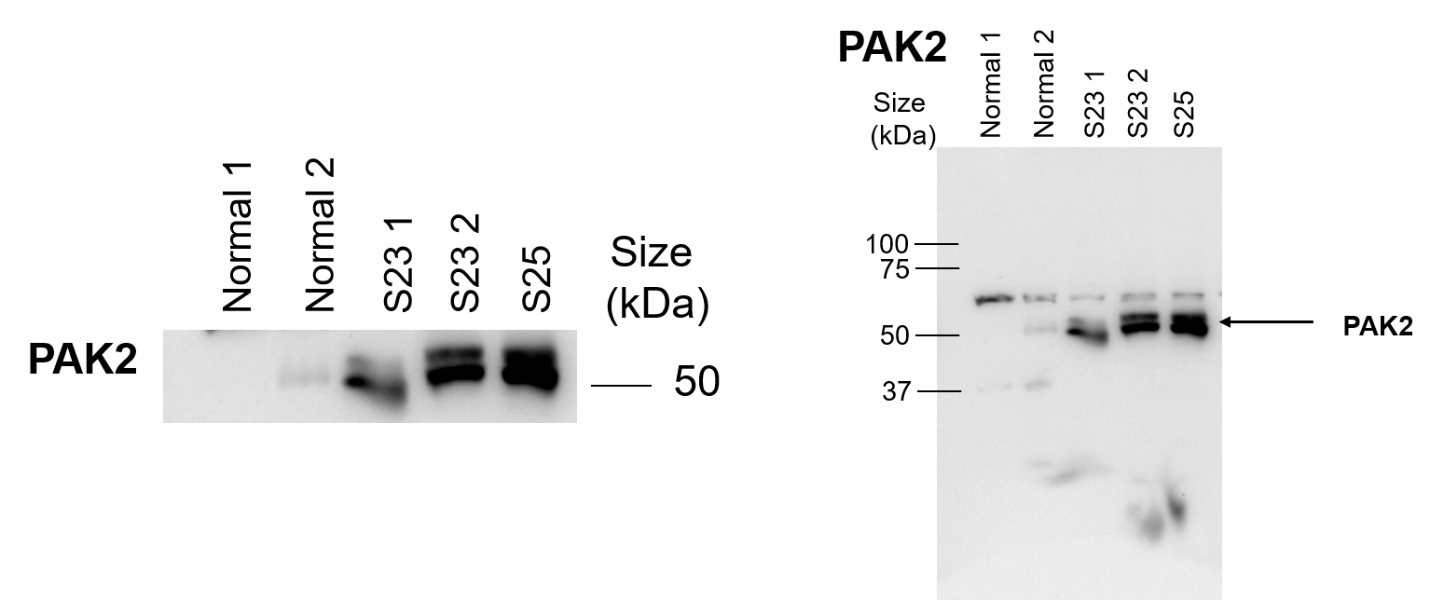


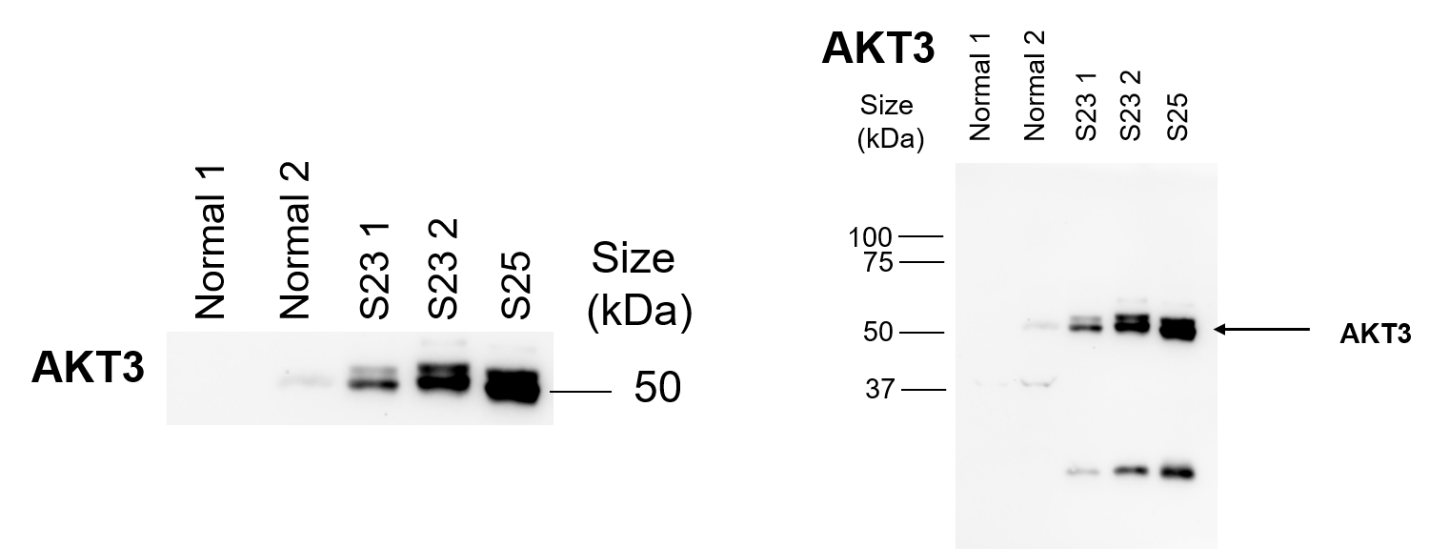


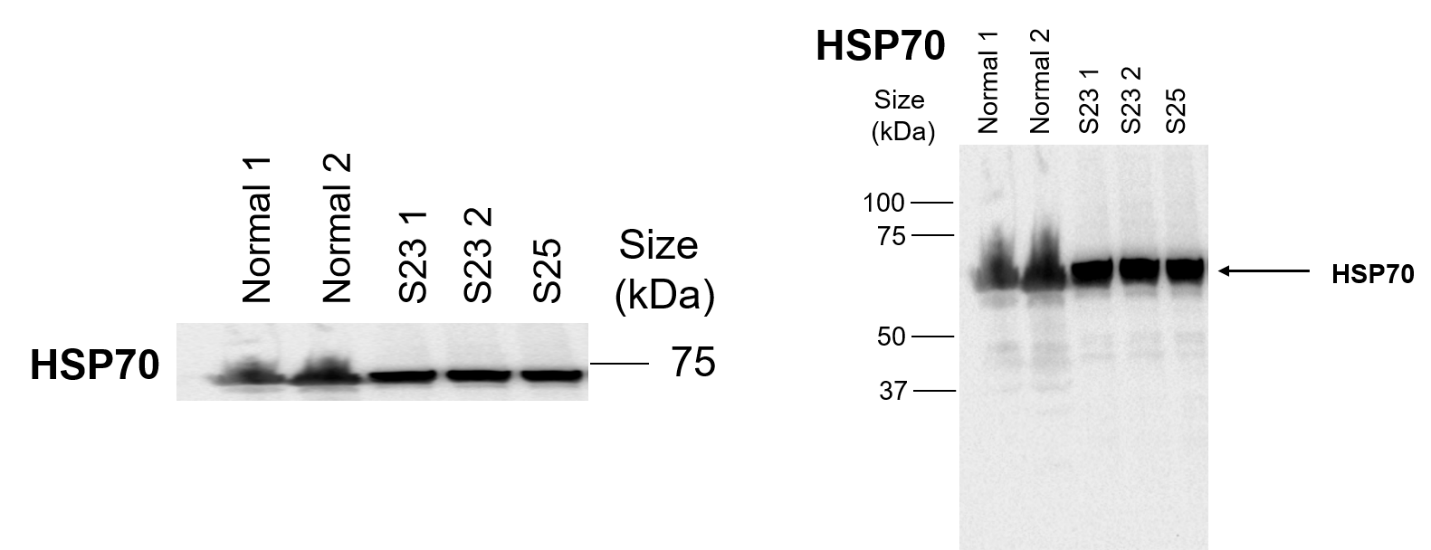

Supplement: Supplementary file 1 — Supplementary Materials [file 41392_2023_1470_MOESM1_ESM.docx]
